# Genetic Factors of the Disease Course After Sepsis: Rare Deleterious Variants Are Predictive

**DOI:** 10.1016/j.ebiom.2016.08.037

**Published:** 2016-09-15

**Authors:** Stefan Taudien, Ludwig Lausser, Evangelos J. Giamarellos-Bourboulis, Christoph Sponholz, Franziska Schöneweck, Marius Felder, Lyn-Rouven Schirra, Florian Schmid, Charalambos Gogos, Susann Groth, Britt-Sabina Petersen, Andre Franke, Wolfgang Lieb, Klaus Huse, Peter F. Zipfel, Oliver Kurzai, Barbara Moepps, Peter Gierschik, Michael Bauer, André Scherag, Hans A. Kestler, Matthias Platzer

**Affiliations:** a Integrated Research and Treatment Center, Center for Sepsis Control and Care (CSCC), Jena University Hospital, Jena, Germany; b Leibniz Institute on Aging – Fritz Lipmann Institute, Jena, Germany; c Institute of Medical Systems Biology, Ulm University, Germany; d 4th Department of Internal Medicine, National and Kapodistrian University of Athens, Athens, Greece; e Department of Anaesthesiology and Intensive Care Therapy, Jena University Hospital, Jena, Germany; f Research group Clinical Epidemiology, CSCC, Jena University Hospital, Jena, Germany; g Department of Internal Medicine, University of Patras, Medical School, Greece; h Institute of Clinical Molecular Biology, Christian-Albrechts-Universität Kiel, Kiel, Germany; i Institute of Epidemiology, Christian-Albrechts-Universität Kiel, Kiel, Germany; j Leibniz Institute for Natural Product Research and Infection Biology – Hans-Knöll-Institute, Jena, Germany; k Septomics Research Center Jena, Leibniz Institute for Natural Product Research and Infection Biology – Hans-Knöll-Institute, Jena, Germany; l Friedrich Schiller University Jena, Jena, Germany; m Institute of Pharmacology and Toxicology, Ulm University Medical Center, Ulm, Germany

**Keywords:** Sepsis, Exome, Rare single nucleotide variation, Population stratification, Classification, Semantic set covering machine

## Abstract

Sepsis is a life-threatening organ dysfunction caused by dysregulated host response to infection. For its clinical course, host genetic factors are important and rare genomic variants are suspected to contribute. We sequenced the exomes of 59 Greek and 15 German patients with bacterial sepsis divided into two groups with extremely different disease courses. Variant analysis was focusing on rare deleterious single nucleotide variants (SNVs).

We identified significant differences in the number of rare deleterious SNVs per patient between the ethnic groups. Classification experiments based on the data of the Greek patients allowed discrimination between the disease courses with estimated sensitivity and specificity > 75%. By application of the trained model to the German patients we observed comparable discriminatory properties despite lower population-specific rare SNV load. Furthermore, rare SNVs in genes of cell signaling and innate immunity related pathways were identified as classifiers discriminating between the sepsis courses.

Sepsis patients with favorable disease course after sepsis, even in the case of unfavorable preconditions, seem to be affected more often by rare deleterious SNVs in cell signaling and innate immunity related pathways, suggesting a protective role of impairments in these processes against a poor disease course.

## Highlights

• Rare SNV load is higher in the Greek vs. German population.
• Subsets of rare deleterious SNVs are predictive for the disease course after sepsis.
• Patients with favorable disease course seem to carry protective deleterious variants in sepsis related pathways.

Sepsis is a life-threatening disease caused by improper response to infection. Only little is known about the role of genetic factors. From > 4000 patients we selected the most extreme cases showing either a favorable or adverse disease course. We determined rare (< 1/200) protein-damaging genetic variants, as they may have a large effect. Using a computational model that includes knowledge on genes we can predict the disease course with > 75% accuracy. Surprisingly, favorable courses can be expected if defense mechanisms are damaged and prevented from overshooting. This underlines the relevance of rare variants for better understanding of sepsis and may offer new treatment options.

## 1 Introduction

According to the new definition (Seymour et al., 2016, Shankar-Hari et al., 2016, Singer et al., 2016), sepsis is a life-threatening organ dysfunction caused by dysregulated host response to infection. Host genetic factors are important for the clinical course (Sorensen et al., 1988, Petersen et al., 2010). Only a limited number of molecular genetic studies in sepsis have been conducted so far - mostly focusing on candidate genes with known methodological challenges (Sutherland and Walley, 2009). Three genome-wide association studies (GWAS) related to sepsis have been performed focusing on different phenotypes (e.g. therapeutic response within a randomized controlled trial (Man et al., 2012) or 28-day mortality (Rautanen et al., 2015, Scherag et al., 2016) and aiming for the identification of common genomic variants. However, rare genomic variants are suspected to contribute to the so-called “missing heritability” (Manolio et al., 2009), and the rare protein-affecting ones - predominantly evolved recently - have a high potential of causing deleterious effects. For example, rare and low-frequency variants with large effects were recently proven to be associated with coronary artery disease (Helgadottir et al., 2016). Furthermore, disease-related genes contain a higher proportion of these deleterious variants than other genes (Fu et al., 2013, Tennessen et al., 2012). Altogether, this suggests that assessment of rare deleterious protein affecting variants is a promising approach for elucidating the genetic component of sepsis. The identified variants can be used as proxies for inferring causality, a key step in identification of novel therapeutic targets.

To assess these variants, whole-exome sequencing (WES) is a successful strategy even for complex diseases like schizophrenia, cardiomyopathy or inflammatory bowel disease (Christodoulou et al., 2012, Loohuis et al., 2015, Norton et al., 2012). WES delivers ten-thousands of variants which subsequently have to be functionally prioritized which is still a critical issue despite the availability of numerous tools (Calabrese et al., 2009, Gonzalez-Perez and Lopez-Bigas, 2011, Li et al., 2009, Reva et al., 2011, Schwarz et al., 2014, Shihab et al., 2013). Remarkably, a unified approach for testing the association between rare variants and phenotypes in sequencing association studies was proposed and evaluated using sepsis-associated acute-lung-injury WES data (Lee et al., 2012).

As sepsis is a complex disease depending on genetic, environmental and live-history traits, we used a classification experiment as proof of principle for the role of rare genetic variants in the disease course. To recruit two classes, we carefully selected the most extreme cases from > 4000 sepsis patients showing either a favorable or adverse disease course. To improve robustness of our approach (i) training and validation cohorts for the classification experiment were selected from different European populations and (ii) different criteria for defining the extremes in the two patient repositories were applied. Altogether, our approach allowed discrimination between the disease courses with high sensitivity and specificity, indicating the relevance of rare deleterious variants for sepsis research and ultimately new clinical applications.

## 2 Materials & Methods

### 2.1 Patients and Samples

Two patient cohorts of different European ethnic background were collected. For the study only patients were considered with at least one sepsis-associated organ failure. Patients with blood cultures yielding isolates of coagulase-negative Staphylococcus spp. or skin commensals were excluded. All subjects or their legal representatives gave written informed consent.

Greek patients were derived from the biobank of the Hellenic Sepsis Study Group which is a collection of biomaterial from patients with sepsis, severe sepsis and septic shock conducted in 65 departments in Greece since May 2006 (www.sepsis.gr). The study protocol is reviewed and approved by the Ethics Committees of the participating study sites (approval 26 June 2006). The selection of eligible patients for WES was done in June 2013 when 3955 patients were enrolled. All patients had a bacteria-positive blood culture. Further selection for extreme clinical phenotypes was done by filtering the patients with two different sets of criteria:

Group A (N = 32): i) age ≥ 18 years; ii) survival after 28 days despite the administration of empirically administered inappropriate antimicrobials. The inappropriateness of antimicrobials was realized when the antibiogram became known;
Group B (N = 27): i) relatively young i.e. age between 18 and 60 years; ii) lack of any comorbidity or other medical condition predisposing to sepsis, iii) critically ill with high mortality rates despite receiving appropriate therapy.

German patients were treated on the same ICU at the University Hospital Jena, Germany (August 2008–May 2011). The study approval was given by the faculty ethics review board (3624-11/12, 2712-12/09, 2160-11/07). All patients presented in clinically bad condition with septic shock resulting from anastomosis insufficiency after major abdominal surgery. Selection of extreme phenotypes from a pool of 120 patients was based on the course of organ dysfunction (measured by Sequential Organ Failure Assessment (SOFA) Scoring) resulting from the same focus of sepsis within a period of five days after sepsis onset:

Group A (N = 5): Patients with fast resolution of organ dysfunction, defined as decreasing SOFA scores of ≥4;
Group B (N = 10): Patients with considerable worsening organ dysfunction, defined as increasing SOFA scores of ≥4.

Although the definitions of sepsis stages of the study protocol were those of 2003, retrospective evaluation showed that all patients met the new Sepsis-3 definition (Seymour et al., 2016, Shankar-Hari et al., 2016, Singer et al., 2016). Detailed description of sepsis patient's characteristics are given in Table S1. Peripheral blood samples were taken from patients under aseptic conditions and kept refrigerated at − 80 °C into an EDTA-coated tube. For all 74 patients, genomic DNAs were prepared from 200 μl blood each using the QIAamp DNA Mini Kit (Qiagen).

WES data of 93 healthy German control individuals were generated at the University Kiel, Germany. These individuals (81/87.1% females; 12/12.9% males; median age: 66; quantiles Q1: 62, Q3: 69) are part of the population-based cohort POPGEN (Nothlings and Krawczak, 2012) and their WES data were recently used as control group data in an early-onset IBD case-control study (Kelsen et al., 2015).

### 2.2 Whole Exome Sequencing

2–3 μg genomic DNA per sepsis patient was fragmented on a Covaris M220 focused ultra-sonicator and exomes were enriched by use of Agilent SureSelect XT Human All Exon V5 + UTRs kit, targeting 74,856,280 bp encompassing the coding sequence and untranslated regions of 20,791 human genes. After sequence capture target enrichment, individual libraries were prepared which were quantified and checked for quality by Agilent High Sensitivity DNA chip. Six libraries were pooled each and sequenced on the Illumina HiSeq2500 platform (RapidRun, 2 × 100 bp Paired End). On average, 5.4 × 10^7^ sequence pairs (10.8 Gb) per sample were generated, corresponding to a 215-fold mean depth of coverage per exome (Table S2). A mean of 21% duplicates was detected. DNAs from control individuals were sequenced at the University Kiel after enrichment using the same kit as for the sepsis patients.

### 2.3 Mapping and Variant Assessment

The Illumina paired-end sequences of the sepsis patients were mapped to the entire human reference genome version GRCh37/hg19 using the Burrows-Wheeler Aligner BWA (Li and Durbin, 2009) with the default settings. Data was processed using the Genome Analysis ToolKit GATK v2.5 (DePristo et al., 2011, McKenna et al., 2010). Regions with alignment gaps were realigned (GATK IndelRealigner), duplicate reads were marked using Picard Tools (http://picard.sourceforge.net) and all aligned read data was subjected to base quality recalibration (GATK BaseRecalibrator). Reads that did not align, or aligned outside of the target regions, were discarded. For the mapped reads we obtained an 87-fold mean depth of coverage, ranging from 40-fold to 155-fold (Table S2). On average, 88% and 80% of all target positions were covered by ≥20 and ≥30 sequence reads, respectively. When extending the calculation by 100 bp up- and downstream of the targeted regions, 75% and 65% of all positions were covered by ≥20 and  ≥30 sequence reads, respectively. Single Nucleotide Variants (SNVs) were called with the GATK UnifiedGenotyper. On average 67,261 SNV calls (84% of all) were marked as “PASS” by the GATK variant quality score recalibration and filtering (GATK VariantRecalibrator and ApplyRecalibration), thereof on average 34,592 SNVs (51% of all PASS SNVs) are located in the exonic regions targeted by the enrichment kit. For these SNVs, the mean ratio of heterozygous variants to those homozygous for the alternate allele is 1.48. The average transition/transversion ratio (Ts/Tv) accounts for 2.73 and was used to calculate the false positive (FP) rate by FP = 1 − (obsTs/Tv − 0.5)/(expTs/Tv − 0.5) with expTs/Tv = 2.8 (Do et al., 2015) corresponding to a false positive rate of 3.1% (Fig. S1). The mean X-chromosomal heterozygosity was calculated with 0.02 for males (N = 51) and 0.29 for females (N = 23) (Fig. S2). These values are similar to those recently calculated from ~ 10,000 exomes by Do et al., reporting a ratio of heterozygous to homozygous SNVs of 1.3–1.8, Ts/Tv of 2.75–2.85 and X-chromosomal heterozygosity of 0.03–0.07 for males and 0.20–0.40 for females (Do et al., 2015). The on-target, GATK passed SNVs exhibited a mean depth of coverage of 63 × and a mean genotyping quality of 92. To assess potential population stratification, we carried out a principle component analysis (PCA) from 258,943 passed SNVs (with SNPdb entry, excluding X/Y and multiallelic variations) using the method of Price et al. with default settings (Price et al., 2006).

**Fig. 1:**
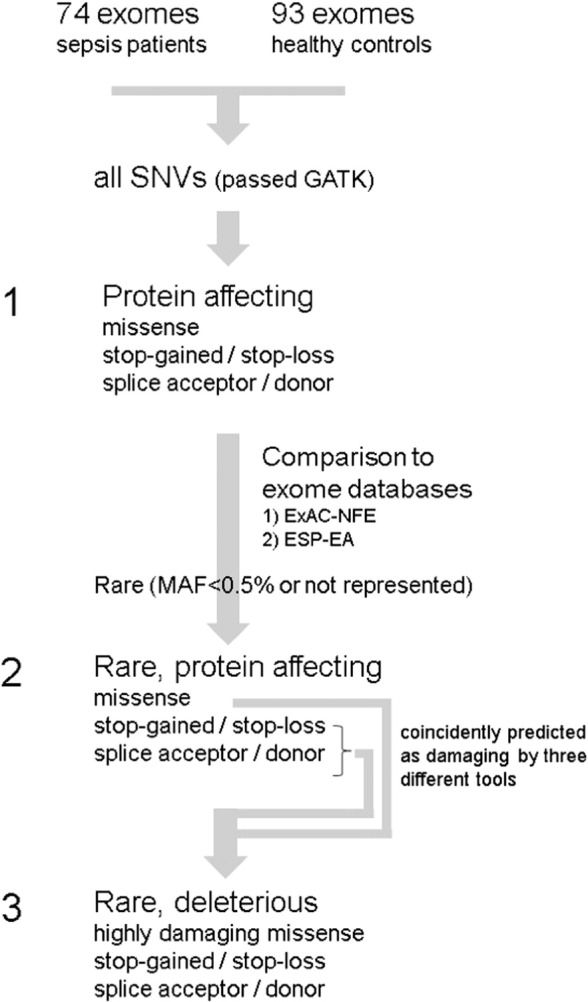
Workflow of variant filtering in three steps. SNV = Single Nucleotide Variant; GATK = Genome Analysis Toolkit; ExAC = Exome Aggregation Consortium, NFE = Non Finnish Europeans, ~ 30,000 exomes; ESP = Exome Sequencing Project NHLBI-ESP, EA = Americans of European Ancestry, ~ 4200 exomes.

All variants assessed by GATK were annotated by the Seattle Sequence Annotation Program (Ng et al., 2009). For further variant's filtering as described in the Results section and Fig. 1, the GATK result vcf-files were parsed by in-house programs. Mapping and variant calling for the control individuals were processed at the University Kiel using the same tools and parameters as described for the sepsis patients (Table S2). The mean depth of coverage for the on-target GATK passed SNVs from controls is lower than for sepsis patients (52 × vs. 63 ×), resulting in a slightly lower mean genotyping quality (84 vs. 92), but the number of SNVs per sample is similar for both cohorts (34,592 vs. 34,201; Table S2).

### 2.4 Identification of Rare Variants

According to our hypothesis that rare variants with intermediate or high phenotypic effect may play an important role in sepsis, we filtered for rare variants. For their identification we explored the currently most comprehensive exome data sets provided by

1) The Exome Aggregation Consortium (ExAC, version 0.3) containing data from ~ 60,000 unrelated individuals of seven ethnical groups and
2) The NHLBI Exome Sequencing Project (ESP, version ESP6500SI-V2) encompassing data from ~ 6500 individuals of two ethnical groups included in studies of heart, lung and blood disorders.

We compared with the allele frequencies of the ExAC non-Finnish European group (ExAC-NFE, ~ 30,000 individuals) and the ESP Americans of European ancestry (ESP-EA, ~ 4200 individuals). Rare variants were defined by MAF < 0.5% in the ExAC-NFE, ESP-EA and the SNP database dbSNP142. Novel variants are those not represented in ESP, ExAC and dbSNP142.

The ratio of novel SNVs accounts to 9.3% with respect to the protein affecting variants (filter 1) and 24.5% for the deleterious SNVs (filter 3). In addition there is an SNV fraction of 2.6% and 5.9% for filter 1 and 3, respectively, representing variants that are represented in at least one of the databases but exhibiting the alternate allele only in non-European populations (Table S3).

### 2.5 Identification of Rare Deleterious Variants

The functional impact of protein affecting variants can considerably differ from harmless (benign) to damaging effects. These effects were evaluated for the rare missense variants by three different programs. PolyPhen-2 (PH) (Adzhubei et al., 2013) uses a naive Bayes classifier to predict the functional importance of an allele replacement by using multiple sequence and structure-based features. The Grantham score (GS) (Grantham, 1974) evaluates the amino acid change effect according to their chemical properties. Finally, SIFT (Kumar et al., 2009) sorts tolerated from non-tolerated changes according to the conservation degree of the amino acid residues. We considered alleles of missense SNVs as damaging if they are coincidentally predicted by these three programs using the following thresholds: PolyPhen-2 PH ≥ 0.904 (probably damaging), Grantham score GS > 100 (> 100, radical and moderately radical), SIFT ≤ 0.05 (not tolerated). Together with the stop-gain/loss and the splice donor/acceptor SNVs, these variants were defined as rare deleterious.

### 2.6 Validation of Selected Variants

#### 2.6.1 Sanger Sequencing

We randomly selected 139 rare heterozygous SNVs for validation using the DNA of 57 patients in which the variants were originally found by WES. PCR primers were selected by Primer 3 (Koressaar and Remm, 2007, Untergasser et al., 2012) and Sanger sequencing of the PCR products was performed on an ABI3730 capillary sequencer using dye-terminator chemistry and the amplification primers. The sequence electropherograms were manually inspected using the Global Alignment Program GAP4.11 and a decision was based on at least one of the two sequencing reads exhibiting an unambiguous signal.

#### 2.6.2 CR1

The protein encoded by *CR1* contains 17 very similar complement control protein modules (CCP) or Sushi domains, differing in only three amino acids. Furthermore, *CR1L*, a paralog of *CR1*, contains parts with high similarity to these domains of *CR1*. GATK identified a heterozygous stop-gain SNV in *CR1* of patient GR-B_01 (chr1:207749025, C>T, not identified in European populations, MAF < 0.01% in ExAC East and South Asian populations) which was assigned to *CR1* exon 20, corresponding to CCP16. However, due to the repetitive structure of this region, it was not sure whether this annotation was correct or should be assigned to *CR1*, exon 12 (CCP9) or *CR1L*, exon 28. We therefore cloned the PCR product used for the Sanger sequencing into pCRTopo4.1, sequenced 36 clones using universal M13f/r primers and were able to discriminate sequence reads with respect to their origin by a sequence motif upstream of the SNV (Fig. S4).

#### 2.6.3 TEAD4

A hemizygous rare deleterious missense SNV in *TEAD4* of sepsis patient GR-A_16 (chr12:3,131,088, rs141718322, C>T, Arg>Cys, MAF 0.14% in Europeans) was investigated for its impact on the Hippo pathway. We synthesized an expression construct harboring the C>T missense mutation and performed cell-based assays to examine possible changes in the TEAD4 protein expression, subcellular localization and interaction with binding partner YAP.

##### 2.6.3.1 Antibodies and Plasmids

For immunoprecipitation, mouse monoclonal anti-FLAG M2 antibody was obtained from Sigma and mouse anti-c-myc antibody was obtained from St. Cruz. For immunoblotting, primary antibodies rabbit anti-DDDDK tag (FLAG tag) and rabbit anti-myc were purchased from Abcam and Millipore/Upstate; the secondary HRP-coupled goat anti-rabbit antibody was purchased from Dako. RK5-myc-TEAD4 was a kind gift from Kunliang Guan (Addgene plasmid #24,638) and pcDNA-FLAG-YAP was a kind gift from Yosef Shaul (Addgene plasmid #18,881) (Levy et al., 2008, Li et al., 2010). The myc-TEAD4 Arg268Cys mutation was generated in accordance with the QuikChange II Site-Directed Mutagenesis Kit protocol, but using *PfuUltra* Hotstart (Agilent Technologies) instead of *PfuUltra* HighFidelity. Mutagenic primer sequences were the following: 5′-CCTACCTCGAAGCCGTGGACATCTGCCAAATCTATG-3′ (forward) and 5′-CATAGATTTGGCAGATGTCCACGGCTTCGAGGTAGG-3′ (reverse). Insertion of the mutation was validated by Sanger sequencing.

##### 2.6.3.2 Cell Culture

HEK293-T cells were maintained in DMEM medium supplemented with 10% FCS (Sigma) in a humidified atmosphere with 5% CO_2_ at 37 °C.

##### 2.6.3.3 Transient Transfection

Transient transfections were performed using jetPEI™ DNA Transfection Reagent (Peqlab) in accordance with the manufacturer's instructions.

##### 2.6.3.4 Co-Immunoprecipitation

HEK293-T cells were grown to 60–70% confluency on 10-cm dishes and transiently transfected as described. 24 h post transfection cells were harvested. Therefore culture dishes were placed on ice; cells were washed with ice-cold PBS and lysed with 1 ml ice-cold Co-IP lysis buffer (50 mM HEPES pH 7.5, 150 mM NaCl, 1 mM EDTA, 1% NP-40 substitute) supplemented with cOmplete Protease Inhibitor and PhosSTOP (Roche), according to (Li et al., 2010). Cell lysates were cleared by centrifugation for 10 min at 10,000 rpm, 4 °C; the supernatant was transferred to new reaction tubes and kept on ice. 500 μl of lysates were incubated with previously prepared antibody/sepharose beads conjugates for 1 h at 4 °C under rotary agitation. Afterwards tubes were centrifuged for 1 min, 4 °C, 2000 rpm and the supernatant was removed from the beads. 1 ml Co-IP wash buffer (50 mM HEPES pH 7.5, 500 mM NaCl, 1 mM EDTA, 1% NP-40 substitute, cOmplete Protease Inhibitor and PhosSTOP was added to the beads, followed by centrifugation for 1 min, 4 °C, 2000 rpm and removal of the supernatant. After 3 repetitive washing steps proteins were eluted from the beads by adding 50 μl SDS loading buffer/0.1 M DTT and subsequent boiling at 95 °C for 10 min. Samples were centrifuged for 1 min, 4 °C, 2000 rpm and the eluted proteins were analyzed by Westernblot. Antibody/sepharose beads conjugates were prepared by incubating either 1 μg (anti-FLAG) or 2 μg (c-myc) of antibodies with 40 μl of GammaBind Plus Sepharose (GE Healthcare) per reaction, for 1 h at 4 °C under rotary agitation, followed by 2 wash steps with Co-IP wash buffer and 1 wash step with Co-IP lysis buffer to equilibrate the antibody/sepharose beads mixture.

##### 2.6.3.5 Westernblot

After SDS-PAGE in 10% polyacrylamide gels, the proteins were transferred onto Nitrocellulose membranes (Carl Roth) by tank blot. Membranes were incubated with blocking buffer (5% fat-free milk (w/v) in TBS-T (0.1% (v/v) Tween-20, 10 mM Tris pH 7.6, 100 mM NaCl)) for 1 h at room temperature followed by incubation with 1:1000-diluted primary antibodies in blocking buffer overnight at 4 °C. After three washes in TBS-T, membranes were incubated with 1:2000-diluted secondary antibody in blocking solution for 1 h at room temperature, and developed and visualized using ECL Western Blotting Substrate (Thermo Scientific) and Amersham Hyperfilm ECL.

### 2.7 Semantic Set Covering Machine

**Fig. 2:**
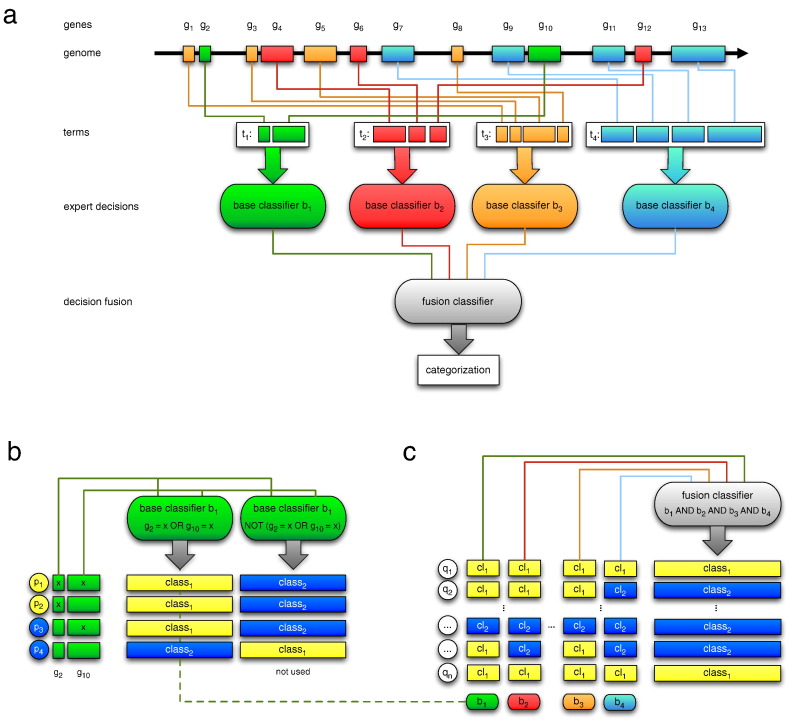
Structure and function of the developed Semantic Set Covering Machine (Sem-SCM). (a) Simplified structure of a trained Sem-SCM. The classifier system derives its prediction by inspecting the SNV status of a set of genes (g_1_,…g_13_). Genes are assigned to base classifiers by semantic terms (t_1_,…,t_4_) that induce a functional or structural grouping like molecular signaling pathways or cellular components. Generally, the same gene can be associated to more than one base classifier. (b) Example of training the Sem-SCM on the genes assigned to base classifier b_1_. Four patients (p_1_, …,p_4_) with known categorization (yellow: class_1_, blue: class_2_) are shown. The base classifier uses a logical disjunction (OR) as a decision rule. The left decision rule will predict class_1_ if g_2_ or g_10_ are affected by a rare deleterious SNV (x) and class_2_ otherwise. The right rule represents its negated form (NOT). In this case the class_1_ will be predicted, if SNVs are detected neither in g_2_ nor in g_10_. Otherwise class_2_ will be assigned. As the application of these rules results in three vs. one correct predictions, the left rule will be utilized. (c) Example of prediction by decision fusion of the base classifiers (logical conjunction AND). It directly operates on the decision rules of the base classifiers (b_1_, …,b_4_). The fusion classifier predicts class_1_ if all base classifiers predict class_1_. Otherwise class_2_ will be assigned. Predictions are shown for patients (q_1_, …,q_n_) not utilized in training.

We have developed a predictor for the disease course of patients after sepsis according to their profiles of rare deleterious SNVs. This predictor model was obtained using a newly developed semantic extension (Sem) of the Set Covering Machine (SCM) (Marchand and Shawe-Taylor, 2003, Kestler et al., 2011), a schematic representation of the Sem-SCM is given in Fig. 2a.

The SCM constructs an fusion decision rule (here a conjunction) of the type

$$\text{IF}\;b_{1}\;\mathrm{AND}\ldots \mathrm{AND}\;b_{N}\;\text{THEN}\;{\mathrm{class}}_{1}\;\text{ELSE}\;{\mathrm{class}}_{2}$$

that can be used to predict a two-group categorization (*class*_*1*_ vs. *class*_*2*_) of newly, so far unseen samples. Here, the class labels correspond to the disease course after sepsis (groups A and B).

Symbols *b*_*1*_, …, *b*_*N*_, denote base classifiers of the sample that can result in either *TRUE* (*class*_*1*_) or *FALSE* (*class*_*2*_, Fig. 2b). A sample is categorized as *class*_*1*_ if all base classifiers result in *TRUE*, otherwise it is categorized as *class*_*2*_ (Fig. 2c).

We have chosen semantic base classifiers that are based on functional and structural groupings of genes (terms). A single term is a set that unites all genes (*g*_*1*_, …, *g*_*S*_) that are associated to a description such as a pathway or GO entry (Fig. 2a). For a single sample, the base classifier *b* results in *TRUE*, if at least one of the corresponding genes (disjunction) is affected by rare deleterious SNVs (x),

$$\text{IF}\;\left(g_{1}=x\right)\;\text{OR}\ldots \text{OR}\;\left(g_{S}=x\right)\;\text{THEN}\;\left(b=\mathrm{TRUE}\right)\;\text{ELSE}\;\left(b=\mathrm{FALSE}\right).$$

A base classifier can alternatively be used in its negated form (NOT, Fig.2b). In this case, the base classifier results in *TRUE*, if no SNV-affected gene is detected.

Training the Sem-SCM means that a set of base classifier *b*_*1*_, …, *b*_*N*_ is selected to form the fusion rule. For our experiments, we utilized predefined groupings from the Molecular Signature Database (Subramanian et al., 2005). The chosen repositories are listed in Table S4.

The strength of SCM training procedure comes from the fact that it constructs a sparse logical conjunction (Marchand and Shawe-Taylor, 2003). As the SCM primarily describes one class (*class*_*1*_) an oversized number of base classifiers will generally lead to a declined sensitivity for *class*_*1*_ and an increased false negative rate. The base classifiers are selected iteratively and depend on previously selected ones (greedy set cover algorithm) (Cormen et al., 1993, Haussler, 1988). A candidate base classifier is chosen in the *i*^th^ iteration if it maximizes the utility function

U=|Q|−p|R|.

Here, |Q| is the number of samples of *class*_*2*_ that are classified correctly by taking the candidate base classifier into account. |*R*| denotes the number of samples of *class*_*1*_ that are misclassified by extending the conjunction. The parameter p can be seen as a weighting parameter. For our experiments it was chosen from the set p ∈ {0.5, 1, 2, ∞}. The second parameter of the training algorithm is the maximal number of base classifiers *s*. It was chosen in the range of *s* ∈ {1, …, 10}. As we have two choices for assigning the class label to the outcome of the decision rule experiments were conducted for both assignments (inv = *TRUE*/*FALSE*).

The performance of the Sem-SCM models was evaluated in leave-one-out cross-validation (LOOCV) experiments. That is, each sample was individually removed from the training process and afterwards used as an independent test sample. The mean performance of the predictor model was used for estimating its generalization ability. All experiments were performed with help of the TunePareto software (Mussel et al., 2012).

## 3 Results

We sequenced the exomes of 59 Greek (GR) and 15 German (DE) patients with validated bacterial sepsis/organ dysfunction according to the new Sepsis-3 definition (Seymour et al., 2016, Shankar-Hari et al., 2016, Singer et al., 2016). Each cohort included two groups of sepsis patients with either favorable (group A) or adverse (group B) disease course after sepsis.

The GR groups were selected from a pool 3955 cases collected by the Hellenic Sepsis Study to represent two qualitatively extremely different phenotypes of medical sepsis patients: group A (GR-A, N = 32) included patients who all survived sepsis, despite unfavorable preconditions given by age, co-morbidities and inappropriate antibiotic therapy, whereas group B (GR-B, N = 27) comprises younger patients without predisposing co-morbidities who normally were not expected to develop sepsis and suffered a fatal outcome in nine cases (33%) irrespective of appropriate antibiotic treatment.

The DE groups represent the quantitative extremes observed among 120 surgical patients at the University Hospital Jena with the same focus of sepsis in respect to the course of organ dysfunction within five days after sepsis onset: group A (DE-A, N = 5): consists of patients with fast resolution of organ dysfunction, defined as decreasing SOFA scores (ΔSOFA = SOFA_Day5_ − SOFA_Day1_; median = − 11, min = − 6, max = − 13), whereas group B (DE-B, N = 10) includes patients with considerable worsening organ dysfunction (+ 4, + 4, + 7) and fatal outcome in three cases (30%, Tables 1 and S1).

### 3.1 Higher Rare SNV Load of Greek Vs. German Patients Is Due to Population Stratification

From the WES data of the 74 sepsis patients SNVs were identified and used for a principle component analysis (Price et al., 2006). The first two components are showing a substantial overlap between either the two ethnic or disease groups, indicating no simple separation due to population stratification effects or clinical phenotype differences, although a cryptic mixture of ancestries may exist in both cohorts (Fig. S3).

To identify potentially sepsis relevant rare variations, the SNVs were filtered in three steps (Fig. 1). In the first step, protein affecting SNVs were selected, encompassing missense, stop–gained (nonsense), stop–loss and splice-acceptor/donor variations, which were filtered in a second step for those with minor allele frequency (MAF) < 0.5%. In the third step, the rare missense SNVs were filtered for high protein damaging effect, which, together with the stop and splice site affecting SNVs are referred to as rare deleterious.

Comparing the amount of SNVs for the different filter steps we identified differences between Greek and German individuals (Table 2). While the number of all SNVs and protein affecting SNVs do not differ significantly, we observed both for the rare protein affecting and rare deleterious SNVs significantly higher amounts for Greek compared to German patients (Wilcoxon rank sum test, p < 0.001). To assess whether these differences represent the population stratification between the two ethnics we used the exome data of 93 healthy Germans (Table S2), the 1000 Genomes Project (1000_Genomes_Project), the Exome Aggregation Consortium (ExAC) and the NHLBI Exome Sequencing Project (ESP). Regarding rare protein affecting and rare deleterious SNVs per individual German patients and controls have a similar SNV load corresponding to non-Finnish Europeans (ExAC-NFE) and Americans of European ancestry (ESP-EA) whereas that of Greek patients corresponds to Southern European populations (Toscani in Italy - TSI, Iberian in Spain - IBS) and Africans (ExAC-AFR; Table S5). This is in agreement with larger heterozygosity in southern compared to northern Europe (Lao et al., 2008, Novembre et al., 2008).

### 3.2 Validation Experiments Confirm Low SNV False Positive Rate

Since validation of WES-assessed variants by Sanger sequencing is still assertively required and regarded as the golden standard for variant detection by NGS. We selected 139 rare heterozygous SNVs identified in 57 patients and performed PCRs followed by Sanger sequencing. In total, in 131 cases (94%) sequencing was successful, confirming the heterozygous state for 127 SNVs while 4 SNVs were found to be homozygous for the reference allele (Table S6). We therefore estimate the fraction of false positive SNVs to 3.0%, which is in agreement with a rate of 3.1% as calculated by the Ts/Tv ratio (Fig. S1).

Two rare deleterious SNVs were evaluated in more detail. First, in depth validation of a heterozygous stop-gain SNV in *CR1* undoubtedly confirmed the GATK annotation in a highly repetitive sequence environment (Fig. S4). The gene encodes for the complement component (3b/4b) receptor 1 (Knops blood group), a transmembrane glycoprotein that prevents accumulation of circulating immune complexes and has an anti-inflammatory effect by inactivation of C3b and C4b. The SNV is likely to result in either truncated translation (1062 amino acids instead of 2039) or nonsense-mediated decay of the respective mRNA leading to a ~ 50% reduced level of CR1 protein in the patient compared to individuals without the variant allele.

Second, a hemizygous missense SNV in *TEAD4* was confirmed and investigated for its impact on the Hippo pathway. TEAD4 and YAP, a transcriptional coactivator, are downstream targets of this pathway, binding to each other by the N-terminal domain of YAP and the C-terminal domain of TEAD4 (Vassilev et al., 2001, Zhao et al., 2008). The Arg268 residue affected in the patient is located in the alpha-1 loop of TEAD. Although this loop is not directly involved in TEAD4-YAP binding (Chen et al., 2010), the Arg268Cys change might have an indirect effect on the TEAD-YAP complex formation. Therefore we expressed the C > T missense allele in cell culture. Co-immunoprecipitation assays and immunofluorescence stainings (data not shown) revealed that neither the binding to YAP nor the subcellular localization was affected (Fig. S5).

### 3.3 Rare Deleterious Variants Are Predictive for the Disease Course After Sepsis

To assess the impact of rare deleterious SNVs on the disease course after sepsis, we performed classification experiments using SNV profiles for training a newly developed Semantic Set Covering Machine (Sem-SCM, Fig. 2). The Sem-SCM preassembles genes that may be affected by the SNVs in predefined and interpretable sets (terms). These terms can for instance be “all genes associated to the signaling pathway Wnt” and can also be utilized as “experts” (base classifiers) for the construction of a decision rule comprised of the individual “expert opinions” (fusion classifier, see Fig. 2c). Training the Sem-SCM is based on rare deleterious variants of the 59 Greek patients and the Molecular Signatures Database (Subramanian et al., 2005) was chosen as source of term sets. Altogether the database comprises seven libraries with 3242 gene sets associated to specific terms, like “Wnt signaling” (Table S4). Optimal parameters were chosen on the training set in a leave-one-out cross-validation (LOOCV) experiment balancing accuracy, sensitivity and specificity.

**Fig. 3:**
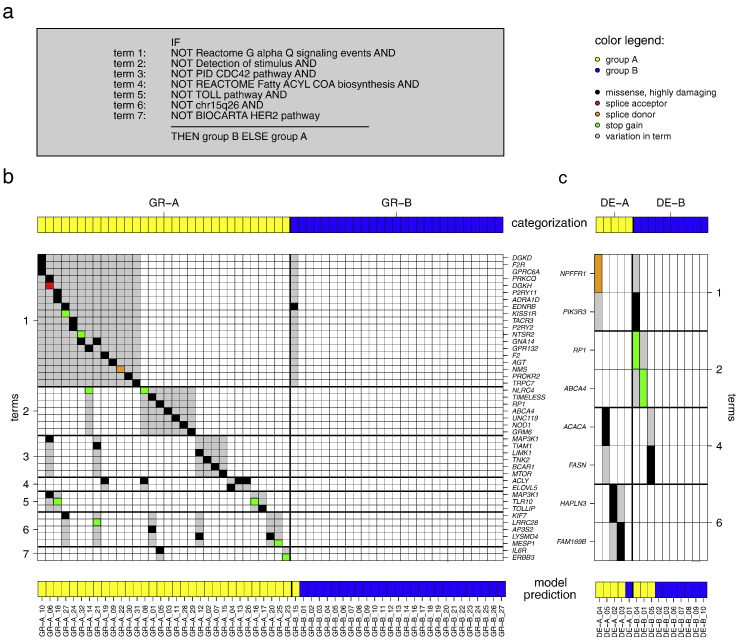
Prediction of the disease course after sepsis onset based on rare deleterious, protein affecting SNVs. Application of the classification model following the rules listed in (a) on 59 Greek (b) and 15 German sepsis patients (c). The samples are shown in columns and sorted according to their class label (32 × GR-A vs. 27 × GR-B and 5 × DE-A vs. 10 × DE-B). First and last row depict sample's categorization and model prediction, respectively. The middle rows show the genes that are affected by SNVs and grouped according to terms. For color code see legend (a).

In the training phase, eleven out of 640 model configurations achieved accuracies > 70% (71.2–76.3%), sensitivities of 51.9–96.3% and specificities of 48.1%–87.5% (Table 3). Ten of these models construct decision rules which are completely based on negated sets (NOT detected) predicting an unfavorable disease course (group B) if rare deleterious SNVs are absent in genes involved in the pathways and/or regions. Six terms are part of the decision in more than two models, including five pathways related to cell signaling and innate immunity, namely the “Gα_q_ signaling”, “detection of stimulus”, “CDC42”, “Toll” and “HER2”. These five pathways encompass 336 genes, of which 36 genes (11%) are affected by rare deleterious SNVs in GR-A in contrast to only one gene in GR-B. The pathway with the most affected genes in A is “Gα_q_ signaling” (20 out of 36, 55%). Remarkably, in nine patients rare deleterious SNVs were found in more than one of the 36 genes. Two genes are affected in two different patients (Table S7). The best model configuration achieved an accuracy of 76.3% (sensitivity for GR-B 77.8%/specificity 75.0%) in the LOOCV (Fig. 3a and b) and proved to be significant in a resampling experiment (p = 0.021, 10,000 relabelings; Supplementary Text).

The performance of the model was further validated on 15 German sepsis patients, which were not included in the training phase. In this experiment, the model correctly classified four out of five DE-A and seven out of ten DE-B patients, corresponding to an accuracy of 73.3% (sensitivity 70.0%/specificity 80.0%, Fig. 2c, Table 3).

## 4 Discussion

To our knowledge, our study is the first reported attempt to estimate the contribution of rare SNVs to the disease course after sepsis. Based on deleterious protein-affecting SNVs, distinction of two different sepsis courses was successful by classification experiments with an SCM-based model. In this investigation no power estimates were performed, as the classification model is not a statistical testing procedure. The quality of the Sem-SCM model is rather characterized in terms of model complexity and overfitting. To ensure here meaningful classification results, minimal decision rules are constructed which are then fused as mixtures of experts. This enables us to stay below the limit given by the theorem of Cover (Cover, 1965) for every base classifier and also uses classifiers with finite Vapnik–Chervonenkis dimension below that of a linear discrimination rule.

A possible causative/functional impact of the identified rare deleterious variants on the sepsis course is supported by two lines of evidence. First, the accuracy of our model with the original dataset was outperformed only by few (2.1%) relabeling experiments. Second, the training process revealed, that the best models with respect to the classification accuracy were based on cell signaling and innate immunity related pathways, namely “Gα_q_ signaling”, “detection of stimulus”, “CDC42”, “Toll” and “HER2”.

In all cases, genes involved in these pathways are more often affected by rare deleterious SNVs in the patients with favorable disease course despite adverse preconditions (group A). This suggests that the putative protein damaging alleles may be protective in case of sepsis, either by loss or gain of gene function, influencing positively the patient's disease management by preventing or limiting overshooting reactions. It also implies that these variants may be of disadvantage, i.e. causing damaging effects, under circumstances not related to sepsis. An example for a protective rare splice donor SNV in anti-fungal immunity and intestinal inflammation has recently been described, resulting in negative regulation of the inflammatory response by CLR-induced CARD9-mediated cytokine production (Cao et al., 2015).

**Fig. 4:**
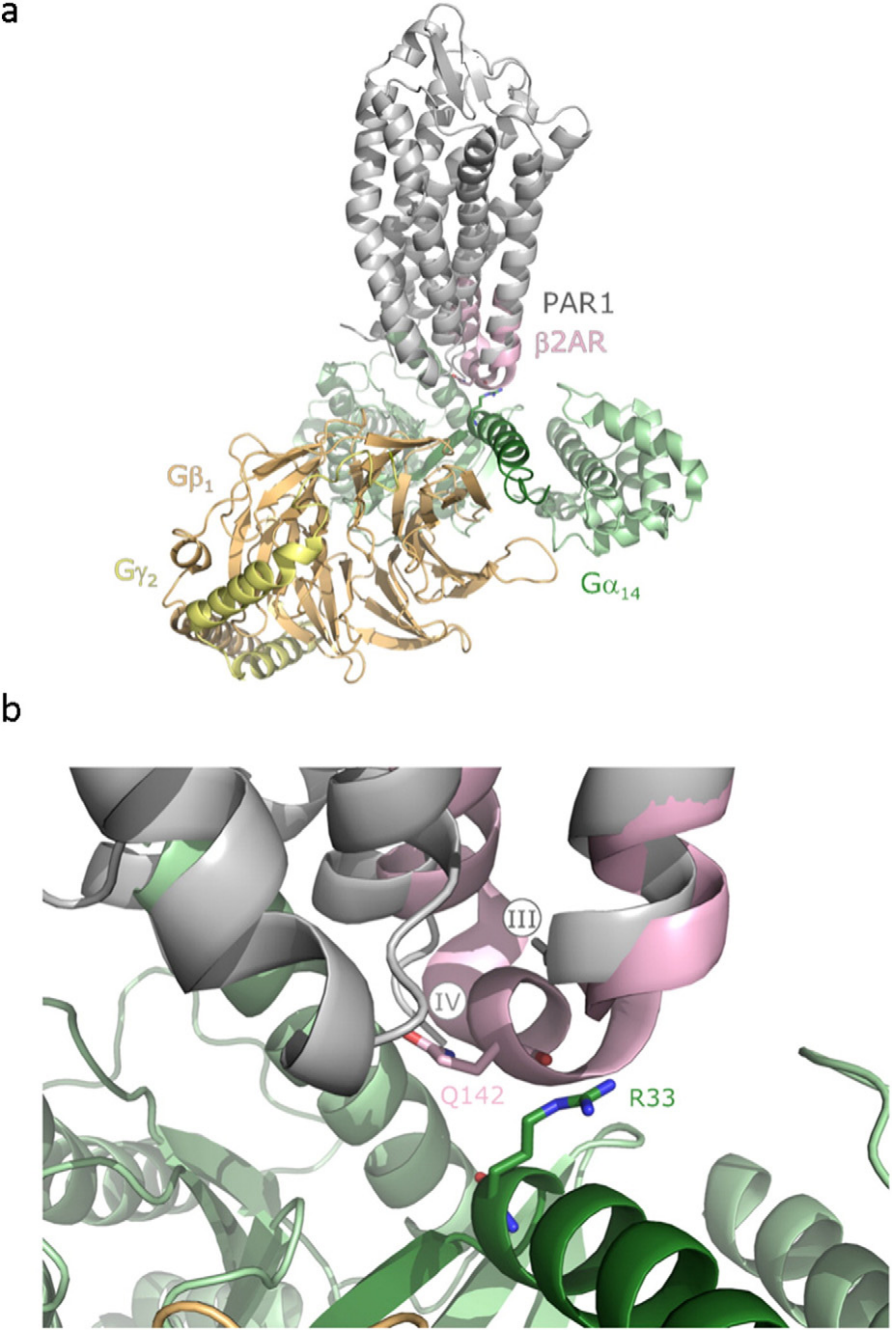
Structural model of the PAR1-Gα_14_ complex indicating a functional impact of amino acid exchange R33C in Gα_14_. (a) The known structure of the human protease-activated receptor 1 (PAR1) (Zhang et al., 2012) was aligned with that of the β_2_-adrenoceptor (β2AR) contained in the quaternary complex between the agonist-bound form of β2AR with heterotrimeric G_s_ (α, βγ) (Chung et al., 2011) using the PyMOL Molecular Graphics System. The structure of the N-terminus of Gα_14_ was predicted with Swiss-Model using the structure of human Gα_q_ as a model (Nishimura et al., 2010) and aligned with the N-terminus of Gα_s_ in the β2AR-G_s_-complex. The structures of Gβ_1_ and Gγ_2_ are those of the β2AR-G_s_-complex. (b) Detailed view of the predicted contact site between PAR1 and the amino terminus of Gα_14_. The junction between transmembrane helices III and IV are missing in the structure of PAR1, presumably due to flexibility of the loop. The C- and N-terminal ends of helices III and IV, respectively, in the structure of PAR1 are marked by circles. In this region, the structure of β2AR is shown in light purple. R^33^ of Gα_14_ is likely to come into very close proximity to the second intracellular loop of PAR1. For example, its distance to Gln^142^ of β_2_-AR, corresponding to L^211^ of PAR1, previously shown to be important for PAR1-G_q_-coupling (Zhang et al., 2012), would be < 3 Å in this model.

The five pathways mentioned above include 336 genes of which 22 are involved in more than one of the five. The most comprehensive is the Gα_q_ signaling pathway including 184 genes, thereof 19 (10%) with rare deleterious SNVs in group A vs. one (0.5%) in group B. Different cellular responses are set in motion by this pathway, mostly, but not exclusively triggered by stimulation of phospholipase C-β (PLCβ) isozymes through receptor-mediated activation of members of the Gα_q_ subfamily of G protein α subunits, including Gα_q_ proper, Gα_11_, Gα_14_, and Gα_15/16_ (Hubbard and Hepler, 2006). Several of the Gα_q_ signaling pathway genes affected in Greek patients encode important mediators of platelet activation, most prominently: *F2* (thrombin) and *F2R* (PAR1), its G_q_-coupled receptor. Thus, platelet activation as part of wound healing might be a key process differing between groups A and B. Some of the rare SNVs are predicted to be functional. For example, the amino acid exchange R33C in Gα_14_ (*GNA14*) is likely to be involved in GPCR-mediated Gα_14_ activation (Fig. 4). Furthermore, S412Y in PAR1 is located in a region of the receptor that is implicated in receptor internalization via phosphorylation- and ubiquitination-dependent sorting (Chen et al., 2011). Some of the genes affected in the G_q_ reactome by rare SNVs have been shown to be involved in sepsis. Thus, PKCθ (*PRKCQ*) has been demonstrated in septic patients to impair chemokine-induced arrest and endothelial transmigration of neutrophils (Berger et al., 2014). G2A (*GPR132*) is activated by commendamide, a metabolite of human commensal bacteria (Cohen et al., 2015) and pretreatment of mice with G2A-specific antibody inhibited lysophosphatidylcholine (LPC)-induced protection from cecal ligation and puncture (CLP) lethality and inhibited the LPC-mediated bactericidal activity of neutrophils in response to *E. coli* ingestion (Yan et al., 2004). Thus, genetic alterations in the G_q_ reactome may also modify the microbe-human-host- relationship. More details are explicated in Supplementary Text.

Our results that pinpoint the Gα_q_ signaling pathway as classificator for the different sepsis courses of patient groups A and B are also supported by a recent GWAS of common variants with respect to the 28-day mortality (Scherag et al., 2016). Among the identified 14 GWAS loci, three are related to Gα_q_ signaling or G-coupled receptors. The top discovery GWAS association signal covers *VPS13A* (related to autophagy) and the 3′ end of the above mentioned *GNA14*. Therefore, both genes are promising functional candidates for the observed association. A second locus highlights *HRH1* (histamine receptor H1), which is part of the Gα_q_ signaling and interleukin receptor SHC pathways. Finally, *GPR12* (G protein-coupled receptor 12) was also identified by the GWAS approach. It has to be noted, though, that the particular GWAS variants in *HRH1* and near *GPR12* were not supported by the GWAS validation data (Scherag et al., 2016).

Although the study appears limited in size, the effort for its enrollment was large, as the investigated extreme disease phenotypes are rare and e.g. the 59 Greek samples were selected from almost 4000 patients. Furthermore, the robustness of our findings is supported by two facts. First, the classification model was trained and validated using samples derived from different ethnical groups. Second, the two groups of sepsis patients with either favorable (group A) or adverse (group B) disease course after sepsis were selected in the two ethnic groups by different criteria. The GR samples were chosen from medical patients to represent two qualitatively extremely different clinical phenotypes, whereas the DE groups represent opposite quantitative extremes among surgical patients. Our findings indicate that careful selection of extremely different clinical phenotypes enables the identification of rare variants underlying complex traits in heterogeneous populations and that respective studies are not limited to populations with reduced allele diversity like Icelanders (Helgadottir et al., 2016).

Our study has not the power to decide which SNVs in which genes – probably in combination or together with more frequent variants – have the assumed protective effect. The proteins encoded by the affected genes, however, are potential therapeutic targets and functional evaluations have to be carried out to narrow down the key players. The functional relation of the identified pathways, namely cellular signaling, pathogen recognition and immune response, underline the relevance of our findings for a better understanding of sepsis and may ultimately lead to improved and personalized treatment options for the disease course.

## Funding Sources

We acknowledge the support by the German Federal Ministry of Education and Research (BMBF) for the Center for Sepsis Control and Care, CSCC, (01EO1002, 01EO1502) and for the Popgen 2.0 Network, P2N, (01EY1103). The research leading to these results received funding from the European Community's Seventh Framework Programme (FP7/2007–2013) under grant agreement n^o^602783, the German Research Foundation (DFG, SFB 1074 project Z1), and the BMBF (Gerontosys II, Forschungskern SyStaR, project ID 0315894A) all to HAK. Andre Franke and Britt-Sabina Petersen are both supported by the DFG Excellence Cluster 306 “Inflammation at Interfaces”.

## Conflict of Interests

EJGB has received honoraria for providing scientific advice to AbbVie, Chicago IL, USA; Astellas Athens, Greece; Biotest AG, Dreieich, Germany; and ThermoFisher Scientific GmbH, Henningdorf, Germany. He has received unrestricted educational funding (paid to the University of Athens) by Biotest AG, Dreieich, Germany; Sanofi SA, Athens, Greece; ThermoFisher Scientific GmbH, Henningdorf, Germany; and by the Seventh Framework European Program HemoSpec. The other authors declare that they have no conflicts of interest.

## Author Contributions

ST, LL, HAK and MP contributed equally to this work. EJGB, MB, OK, KH and MP created the study concept and design. EJGB, CG, and CS selected the patients and provided the blood samples. ST, LL, FS, MF, LRS, FS and AS performed data acquisition and analyses. BSP, AF, and WL provided the data of the German control samples. SS, PFZ, BM, and PG carried out functional validations. MP and HAK supervised and guided the study. ST, LL, HAK, and MP wrote the manuscript, all other authors participated in its finalization.

## Figures and Tables

**Table 1:** Characteristics of sepsis patients (for individual data see Table S1).

	Greek (GR), N = 59^a^		German (DE), N = 15^b^	
Group	A	B	A	B
Number	32	27	5	10
Deaths within 28 days	0	9 (33%)	0	3 (30%)
Men	22 (69%)	21 (78%)	4 (80%)	4 (40%)
Women	10 (31%)	6 (22%)	1 (20%)	6 (60%)
Age [median (Q1;Q3)^c^]	78.0 (65.0; 82.0)	47.0 (33.0; 53.0)	69.0 (53.0; 70.5)	64.5 (51.2; 72.7)
Sepsis focus				
– Bacteremia	9	16	0	0
– Acute pyelonephritis	14	2	0	0
– Pneumonia	5	4	0	0
– Cholangitis	2	0	0	0
– Soft tissue infection	1	0	0	0
– Abdominal infections	1	2	5	10
– Peritonitis	0	2	0	0
– Unknown	0	1	0	0
APACHE II [median (Q1;Q3)]^d^	17.0 (13.0; 20.5)	18.0 (14.7; 26.0)	27.0 (15.0; 30.0)	22.0 (18.8; 26.3)
SOFA [median (Q1,Q3)]^d^	5.0 (4.0; 7.5)	9.0 (6.0; 14.0)	11.0 (7.0; 20)	10.0 (6.0; 12.3)
Failing organs [median (range)]	1 (1–4)	2 (1–5)	4 (2–5)	4 (2–6)
Patients with ALI^e^	3	0	1	2
Patients with ARDS^f^	11	16	3	6
Pathogen identified	32 (100%)	27 (100%)	3 (40%)	7 (70%)
– Gram-positive infection only	4	3	0	1
– Gram-negative infection only	26	22	1	3
– Two gram-negative pathogens	1	1	1	0
– Gram-positive and -negative	1	1	0	2
– Fungi	0	0	1	1

a Medical patients.

b Surgical patients.

c Q: quantile.

d Score at sepsis onset.

e ALI: acute lung injury.

f ARDS: acute respiratory distress syndrome.

**Table 2:** Variants identified from sepsis patients and controls.

Filter step	SNVs	Greek		German			
		Sepsis				Controls	
		GR (N = 59)	Avg^a^	DE (N = 15)	Avg^a^	DE (N = 93)	Avg^a^
	All	289,521	67,199.8	190,671	67,499.9	278,893	67,831.5
1	Protein affecting	45,261	8581.2	25,729	8513.3	48,094	8508.6
2	**Rare**^b^**protein affecting**	**17,726**	**302.8**^d^	**4403**	**251.1**	**18,218**	**237.1**
	Missense	17,236	294.1	4303	244.3	17,627	230.0
	Stop and splice	490	8.7	100	6.9	591	7.1
3	**Rare**^b^**deleterious**	**2211**	**40.3**^d^	**477**	**32.9**	**2615**	**33.2**
	Missense, Damaging^c^	1721	31.6	377	26.0	2024	26.1
	Stop-gain (nonsense)	322	5.8	67	4.5	392	4.6
	Stop-loss	17	0.3	5	0.3	9	0.1
	Splice-acceptor	75	1.3	13	1.0	87	1.1
	Splice-donor	76	1.3	15	1.0	103	1.3

a Average per sample.

b MAF < 0.005 in ExAC-NFE and ESP-EA.

c Coincidently predicted to be damaging by PolyPhen, Grantham score and SIFT.

d Significantly higher for GR vs. DE patients (Wilcoxon rank sum test, p < 0.001).

**Table 3:** Leave-one-out-cross validation (LOOCV) models with accuracies > 75% for the classification of 59 Greek sepsis patients (top) and application of the two best models to 15 German patients (bottom).

Parameters^a^	Model^a^	Acc^a^	Sens^a^	Spec^a^	Decision	Decision rule
Meta = all, inv = Y, s = 10, p = 2	1	0.763	0.778	0.750	Group B	IF NOT reactome G alpha Q signaling events AND NOT detection of stimulus AND NOT PID CDC42 pathway AND NOT reactome fatty acyl CoA biosynthesis AND NOT biocarta toll pathway AND NOT chr15q26 AND NOT biocarta HER2 pathway
Meta = all, inv = Y, s = 2, p = 2	2	0.763	0.963	0.594	Group B	IF NOT reactome G alpha Q signaling events AND NOT detection of stimulus
Meta = all, inv = Y, s = 7, p = 2	3	0.763	0.778	0.750	Group B	IF NOT reactome G alpha Q signaling events AND NOT detection of stimulus AND NOT PID CDC42 pathway AND NOT reactome fatty acyl CoA biosynthesis AND NOT biocarta toll pathway AND NOT chr15q26 AND NOT biocarta HER2 pathway
Meta = all, inv = Y, s = 8, p = 2	4	0.763	0.778	0.750	Group B	IF NOT reactome G alpha Q signaling events AND NOT detection of stimulus AND NOT PID CDC42 pathway AND NOT reactome fatty acyl CoA biosynthesis AND NOT biocarta toll pathway AND NOT chr15q26 AND NOT biocarta HER2 pathway
Meta = all, inv = Y, s = 9, p = 2	5	0.763	0.778	0.750	Group B	IF NOT reactome G alpha Q signaling events AND NOT detection of stimulus AND NOT PID CDC42 pathway AND NOT reactome fatty acyl CoA biosynthesis AND NOT biocarta toll pathway AND NOT chr15q26 AND NOT biocarta HER2 pathway
Meta = all, inv = Y, s = 6, p = 2	6	0.746	0.778	0.719	Group B	IF NOT reactome G alpha Q signaling events AND NOT detection of stimulus AND NOT PID CDC42 pathway AND NOT reactome fatty acyl CoA biosynthesis AND NOT biocarta toll pathway AND NOT chr15q26
Meta = react, inv = Y, s = 2, p = 2	7	0.729	0.963	0.531	Group B	IF NOT reactome G alpha Q signaling events AND NOT reactome triglyceride biosynthesis
Meta = react, inv = Y, s = 3, p = 2	8	0.729	0.963	0.531	Group B	IF NOT reactome G alpha Q signaling events AND NOT reactome triglyceride biosynthesis AND NOT reactome amine compound SLC transporters
Meta = kegg, inv = N, s = 4, p = Inf	9	0.712	0.906	0.481	Group A	IF NOT kegg inositol phosphate metabolism AND NOT kegg amyotrophic lateral sclerosis ALS AND NOT kegg long term potentiation AND NOT kegg butanoate metabolism
Meta = all, inv = Y, s = 3, p = 2	10	0.712	0.852	0.594	Group B	IF NOT reactome G alpha Q signaling events AND NOT detection of stimulus AND NOT PID CDC42 pathway
Meta = kegg, inv = Y, s = 3, p = 1	11	0.712	0.519	0.875	Group B	If kegg MAPK signaling pathway AND NOT kegg cysteine and methionine metabolism AND NOT kegg acute myeloid leukemia


Parameters^a^	Model	Group	Predicted as A	Predicted as B
Meta = all, inv = Y, s = 10, p = 2	1	DE-A (N = 5)	4	1
		DE-B (N = 10)	3	7
Meta = all, inv = Y, s = 2, p = 2	2	DE-A (N = 5)	1	4
		DE-B (N = 10)	2	8

a Acc: accuracy, Sens: sensitivity, Spec: specificity, meta: source of meta-information, inv: inversion of class labels (Y/N), s: maximal number of base classifiers (1–10), p: weighting parameter (0.5, 1, 2, ∞).

## Appendix A Supplementary Data

Supplementary text and figures. Image 1

Supplementary tables. Image 2
