# Supplementary material for: Genetic Factors of the Disease Course After Sepsis: Rare Deleterious Variants Are Predictive
Source: eBioMedicine. 2016 Sep 15;12:227–38. doi: 10.1016/j.ebiom.2016.08.037 (PMC5078585; doi:10.1016/j.ebiom.2016.08.037)
Supplement: Supplementary file 1 — Supplementary text and figures. [file mmc1.docx]

**Supplementary Text**

**Resampling experiments**

For assessing the flexibility of the prediction model, we repeated the LOOCV experiment for 10,000 relabeled versions of the GR dataset assigning the group labels A and B randomly to the Greek samples. For each of these datasets, the model was retrained and evaluated in a LOOCV allowing the measurement of the model’s ability to learn arbitrary dichotomies. If a large number of randomly labeled datasets would result in higher accuracies than for the original one, the model would be too flexible. However, in over 10.000 repetitions only 2.1% of the randomly labeled datasets could exceed the original accuracy.

**Functional aspects with regard to the Gα_q_ signaling pathway**

Gα_q_ proteins mostly, but not exclusively, act by mediating the stimulation of phospholipase C-β (PLCβ) isozymes following their own activation through specific G-protein-coupled receptors (GPCRs). PLCβ stimulation leads to enhanced hydrolysis of phosphatidylinositol 4,5-bisphosphate (PtdIns*P*_2_), followed by increases in the intracellular concentrations of Ca^2+^ and diacylglycerol {Hubbard, 2006 #183}. All four members of the Gα_q_ family are coupled to PLCβ activation, yet, are functionally diverse and some of their cellular actions are independent of inositol lipid signaling. In addition to thrombin and its G_q_-coupled receptor PAR1, other Gα_q_ signaling pathway genes affected in Greek patients are involved in platelet activation or regulation thereof, such as PKCθ {Cohen, 2011 #185}, EDNRB {Hamroun, 1998 #190}, KISS1R {Mezei, 2015 #186}, and TACR3 {Gibbins, 2009 #187}. Mice deficient in Gα_q_ proper exhibit increased bleeding times and are protected from thromboembolism {Offermanns, 1997 #152}. Thus, platelets are likely candidates for cell types mediating the difference between groups A and B. Gα_14_ is primarily expressed in kidney, liver, and lung, and is also present in many other tissues; it is unknown, however, whether it is present in platelets. Within the Gα_q_ subfamily, Gα_14_ has a relatively limited receptor coupling specificity, and is thus expected to be activated by a wider array of G-protein-coupled receptors (GPCRs) than Gα_q_ and Gα_11_ {Hubbard, 2006 #183}. This is consistent with the association of several G_q_-family-coupled receptors with Gα_14_ in the "G_q_ reactome". Recently, PAR1 (F2R) was reported to functionally interact with Gα_14_ {McCoy, 2010 #176}.

While no three-dimensional structural of a receptor (e.g. PAR1)-heterotrimeric G_14_-complex is currently available, a structural model can be constructed using the known three-dimensional structures of human PAR1 {Zhang, 2012 #173}, human hetrotrimeric G_q_ {Nishimura, 2010 #195}, and of the of the complex between the agonist-bound human β_2_ adrenoceptor with heterotrimeric G_s_ (α, βγ) as a template {Chung, 2011 #172}. In this model, R33 of Gα_14_ is likely to come into very close proximity (≤ 3 Å) to the second intracellular loop of PAR1, e.g. to residue Leu^211^, which was previously shown to be important for PAR1-G_q_-coupling {Zhang, 2012 #173}. Hence, the Gα_14_ R33C mutation identified in this study may have functional consequences for the receptor-mediated G_14_ activation. Of note, alterations in the expression and structure of Gα_14_ have previously been shown to be associated with defective regulation of the vascular tone {Zhao, 2014 #196; Kohara, 2008 #188; Abdul-Salam, 2010 #189}.

**Supplementary Figures**

**
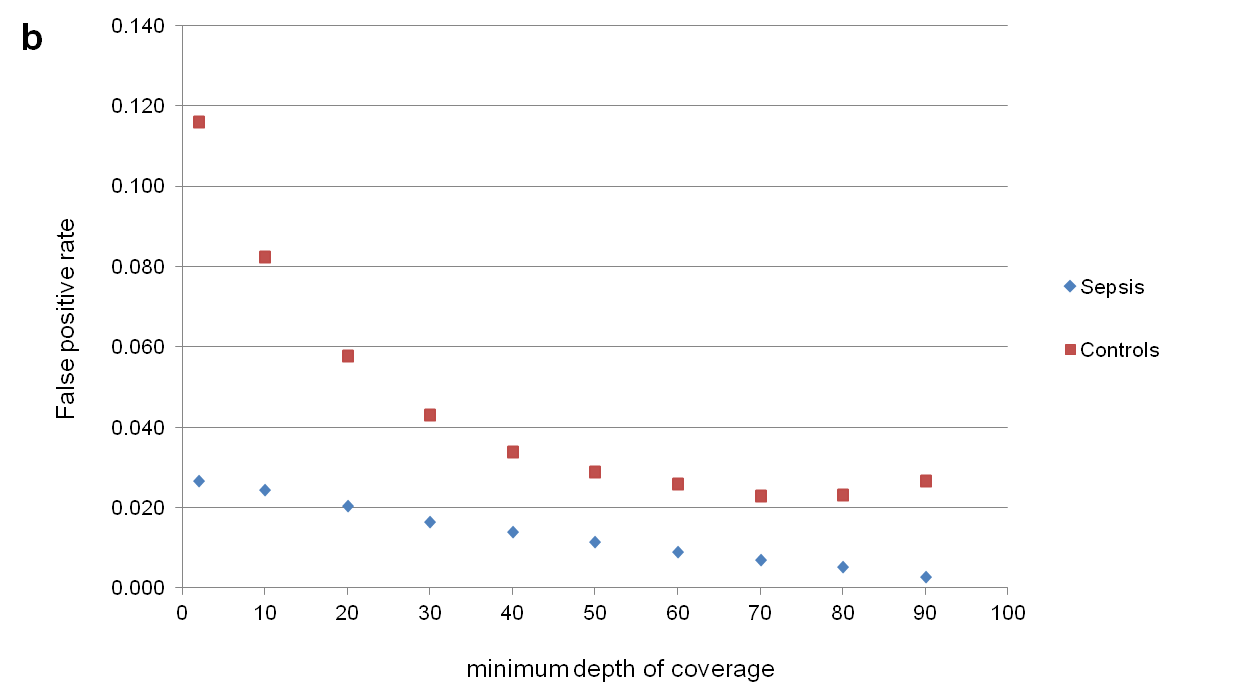

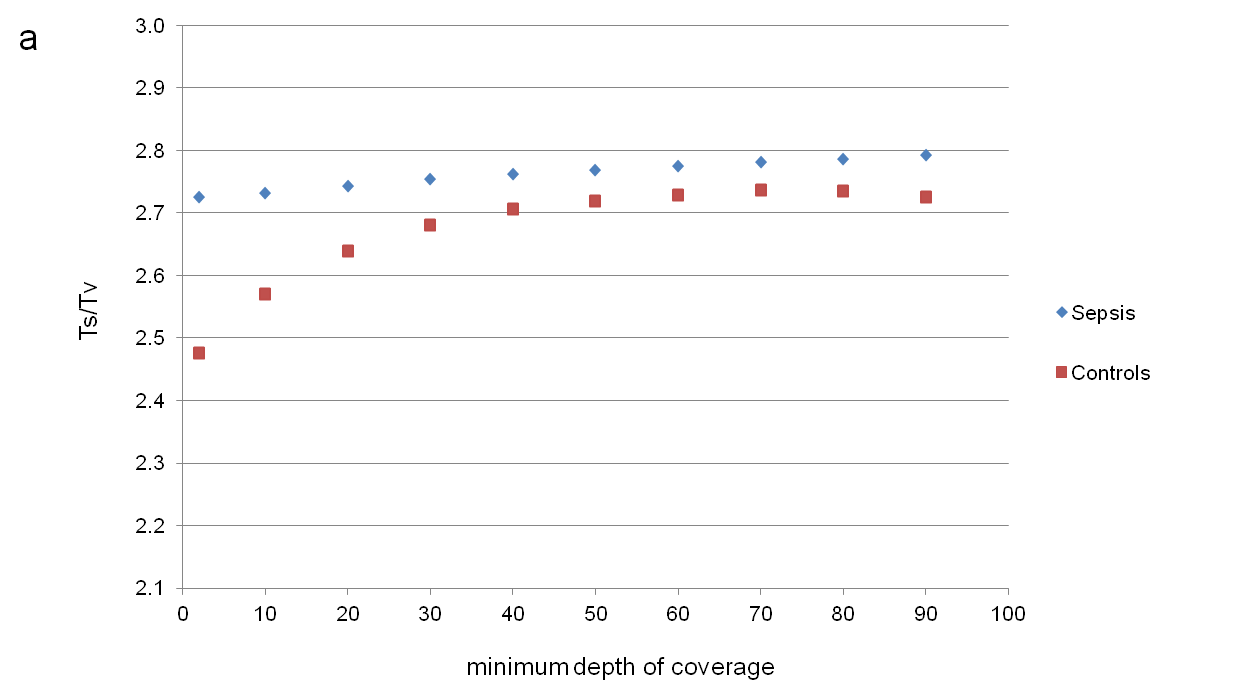
**

**Fig. S1.** Transversion/Transition (Ts/Tv) ratios (**a**) and false positive (FP) rate estimation (**b**) for SNVs of sepsis patients and German controls in the regions targeted by the Agilent SureSelect XT Human All Exon V5 + UTRs kit .

**
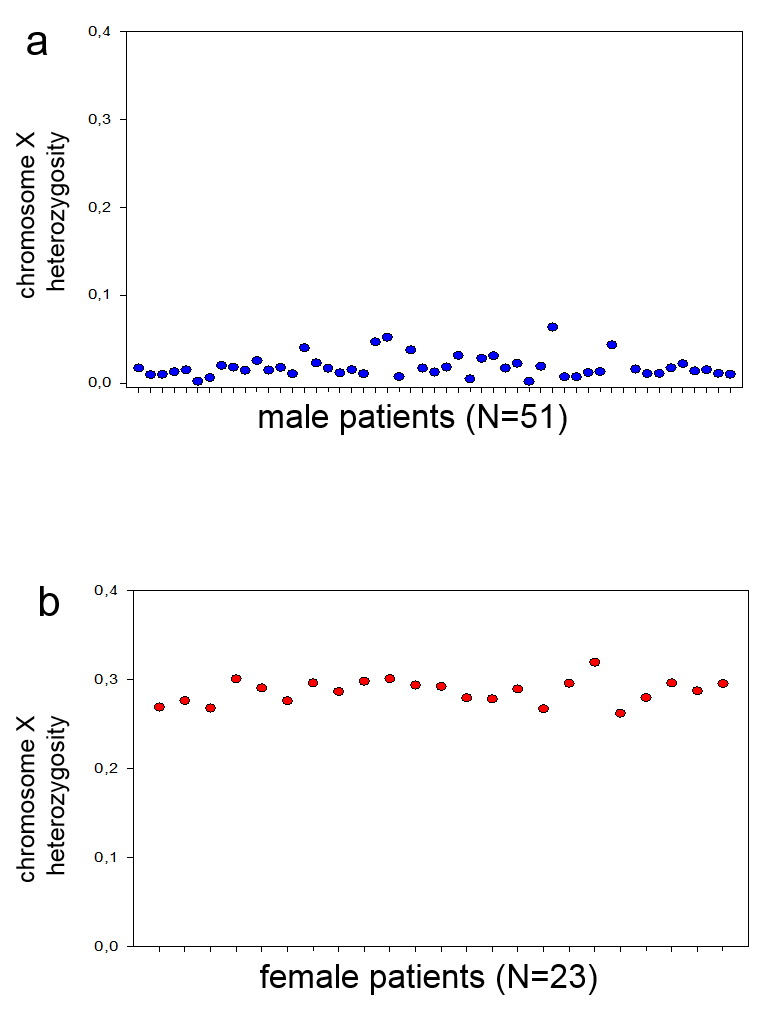
**

**Fig. S2.** X-chromosomal heterozygosity for male (a) and female (b) sepsis patients.


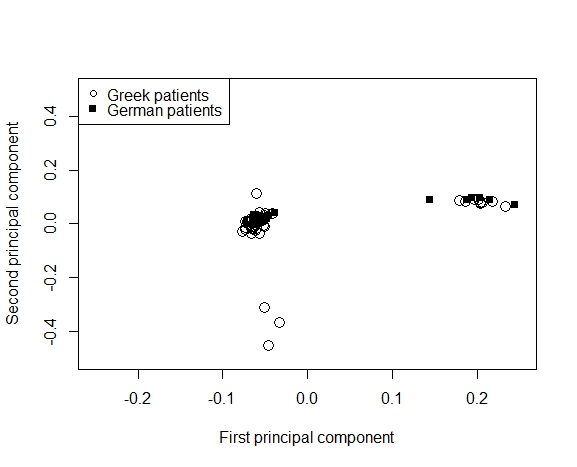

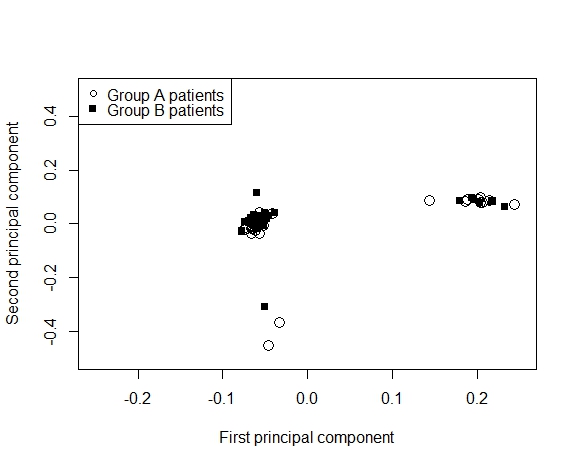


a

b

**Fig. S3.** Principal component analysis of the WES SNV data from the 74 sepsis patients.

The first two principal components were plotted and individuals highlighted (**a**) by country and (**b**) by patient group.

**
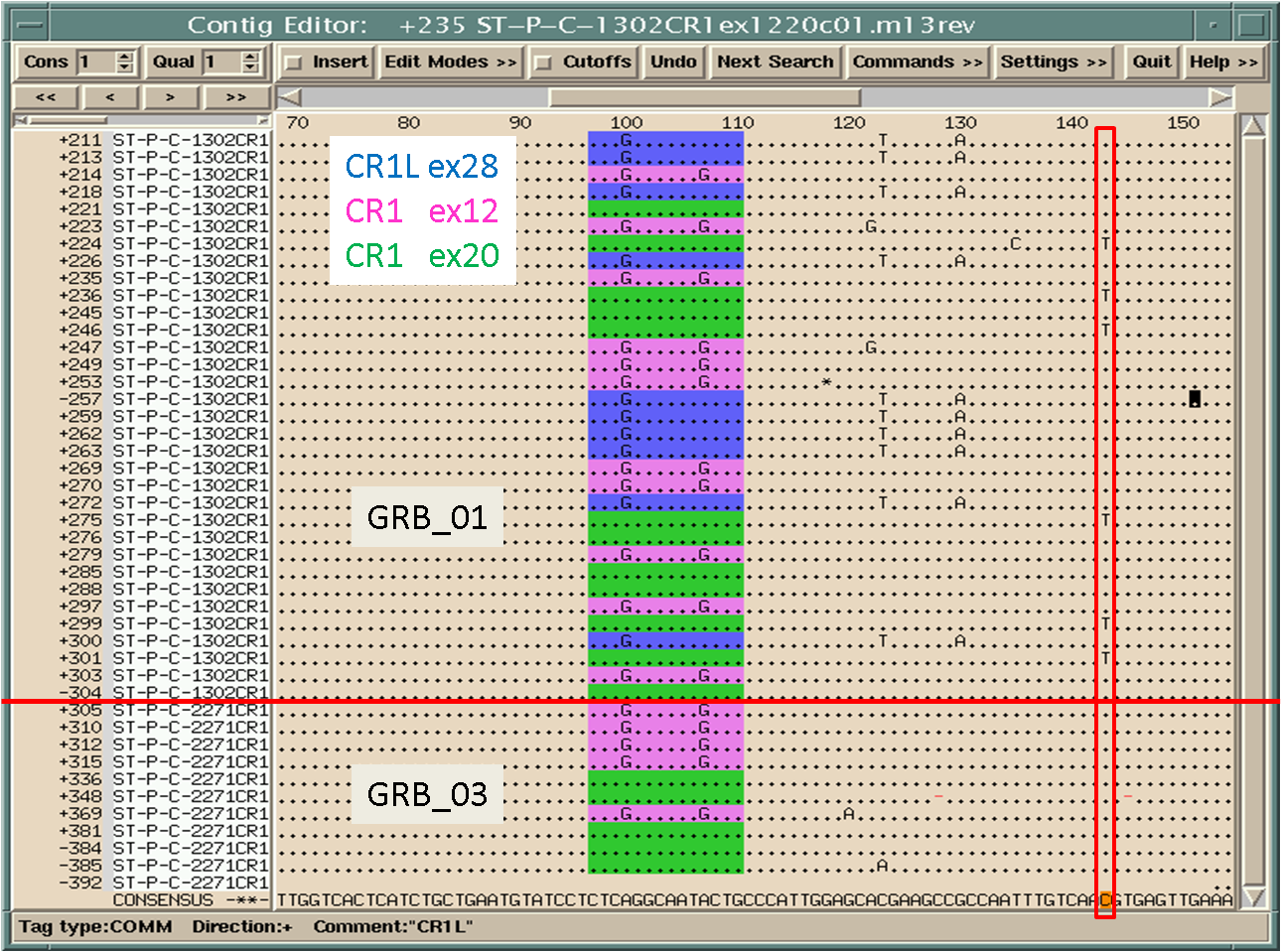
**

**Fig. S4.** Validation of a heterozygous stop-gain SNV in *CR1.*

Sanger sequencing reads can be discerned by the highlighted sequence motifs with respect to their origin (exon). Colours: magenta-CR1 exon 12, green-CR1 exon 20, blue-CR1L exon 28. The alternate allele T at the position to be validated is exclusively represented by reads from CR1, exon 20 of patient GRB_01 (red frame), assigning the protein truncation undoubtedly to CR1, CCP16.

**
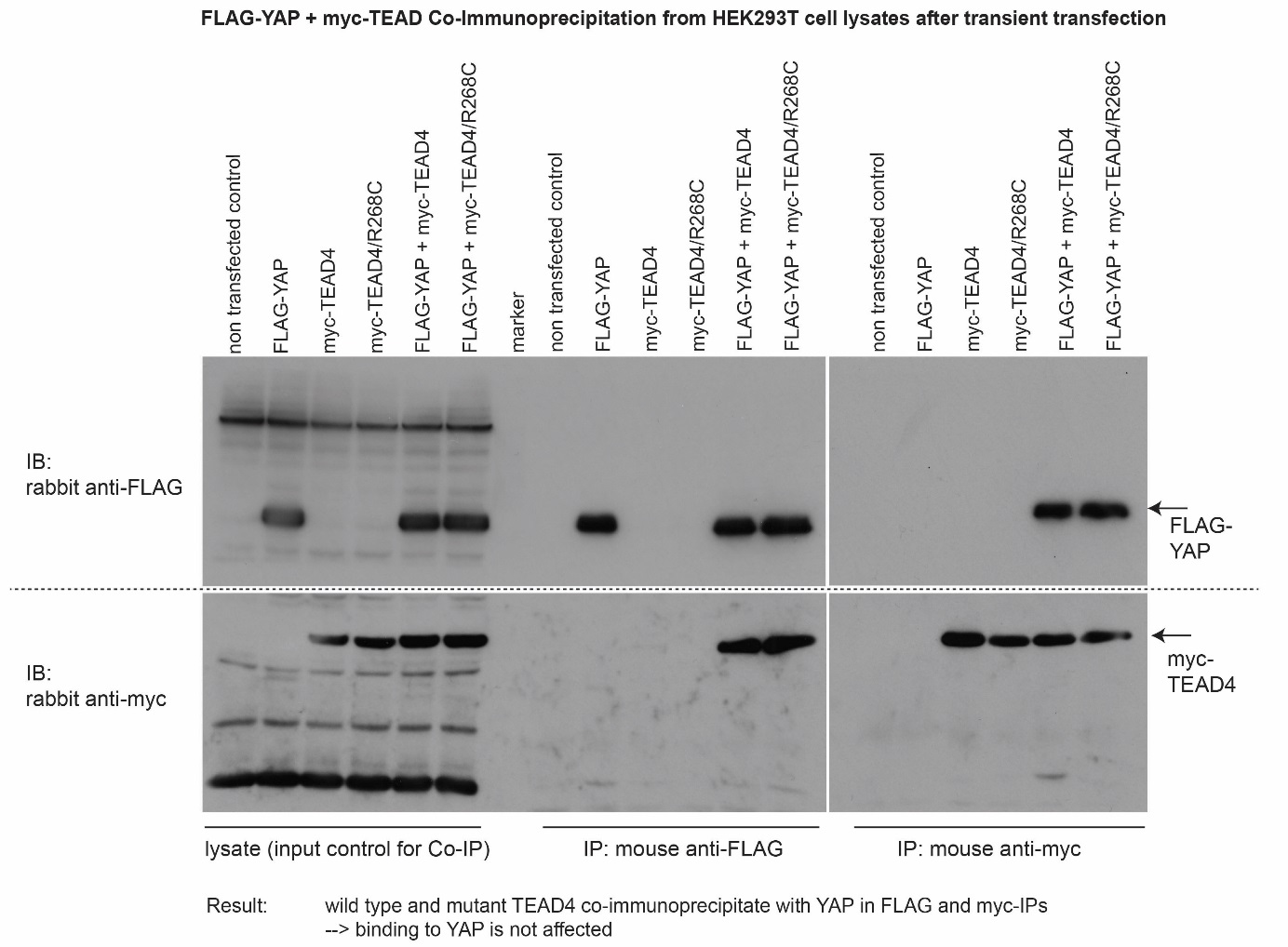
**

**Fig. S5.** The Arg268Cys mutation in TEAD4 has no impact on its binding to the YAP protein.

HEK293-T cells were transfected as indicated, protein expression and precipitated proteins were analyzed by Westernblot. 2% of Co-IP lysates served as input controls, shown on the left side. The wildtype and mutant form of TEAD4 were co-precipitated with FLAG-YAP (middle part), and FLAG-YAP co-precipitated with both, wildtype and mutant myc-TEAD4, in reciprocal Co-IPs (right part).

**Supplementary Tables**

**Table S1**

Detailed description of sepsis patient's characteristics.

(see SupplementaryTables.xls)

**Table S2**

Sequencing, mapping and SNV calling data for sepsis patients (N=74) and controls (N=93).

(see SupplementaryTables.xls)

**Table S3**

Fractions of novel and non-European SNVs identified in the 74 sepsis patients.

| **Filter** | **Category** | **SNVs** | **Novel ^a^** | Fraction | **Non-European** ^b^ | Fraction |
| --- | --- | --- | --- | --- | --- | --- |
| **1** | protein affecting | 49,958 | 4,623 | 0.093 | 1,303 | 0.026 |
| **2** | rare ^c^ protein affecting | 20,951 | 4,603 | 0.220 | 988 | 0.047 |
| **3** | rare deleterious ^b^ | 2,644 | 648 | 0.245 | 155 | 0.059 |

^a^ SNV not represented in ExAC, ESP or dbSNP

^b^ SNV represented in ExAC, ESP and/or dbSNP but alternate allele identified only in non-European populations

^c^ MAF<0.005 in ExAC-NFE and ESP-EA

^d^ missense SNVs coincidently predicted to be damaging by PolyPhen, Grantham score and SIFT, stop and splice site affecting SNVs

**Table S4**

Predefined definitions downloaded from the Molecular Signature Database ^1^ and used for classification experiments.

| **Collection of gene sets** (Name and Citations) | **Number of gene sets** |
| --- | --- |
| BioCarta ^2^ | 217 |
| Gene Ontology (GO) ^3^ | 1,454 |
| Kyoto Encyclopedia of Genes and Genomes (KEGG) ^4^ | 186 |
| Pathway Interaction Database (PID) ^5^ | 196 |
| Positional Gene Sets ^6,7^ | 326 |
| Oncogenic Signatures ^1^ | 189 |
| Reactome ^8^ | 674 |

1. Subramanian, A. et al. Gene set enrichment analysis: a knowledge-based approach for interpreting genome-wide expression profiles. *Proc Natl Acad Sci U S A* 102, 15545-50 (2005).

2. Strausberg, R.L., Buetow, K.H., Emmert-Buck, M.R. & Klausner, R.D. The cancer genome anatomy project: building an annotated gene index. *Trends Genet* 16, 103-6 (2000).

3. Ashburner, M. et al. Gene ontology: tool for the unification of biology. The Gene Ontology Consortium. *Nat Genet* 25, 25-9 (2000).

4. Kanehisa, M. & Goto, S. KEGG: kyoto encyclopedia of genes and genomes. *Nucleic Acids Res* 28, 27-30 (2000).

5. Schaefer, C.F. et al. PID: the Pathway Interaction Database. *Nucleic Acids Res* 37, D674-9 (2009).

6. Schuler, G.D. et al. A gene map of the human genome. *Science* 274, 540-6 (1996).

7. Wright, M.W. & Bruford, E.A. Human and orthologous gene nomenclature. *Gene* 369, 1-6 (2006).

8. Croft, D. et al. Reactome: a database of reactions, pathways and biological processes. *Nucleic Acids Res* 39, D691-7 (2011).

**Table S5**

Average of SNVs per individual for the different ethnics, separated by SNV class and filter steps according to Fig.1.

| **Cohort / DataBase** | **Ethnics** | **N** | **Rare missense** | **Rare 3fold damaging** | **Rare stop** | **Rare splice** | **Rare protein affecting** | **Rare deleterious** |
| --- | --- | --- | --- | --- | --- | --- | --- | --- |
| **Sepsis** | Greek (GR) | 59 | 294.07 | 31.61 | 6.08 | 2.61 | 302.76 | 40.31 |
| **1000G** | Iberian in Spain (IBS) | 107 | 305.37 | 32.27 | 6.01 | 3.35 | 314.73 | 41.63 |
| **1000G** | Toscani in Italy (TSI) | 107 | 308.14 | 34.16 | 6.25 | 3.89 | 318.28 | 44.30 |
| **ExAC** | African (AFR) | appr. 8,000 | 310.93 | 29.01 | 6.49 | 3.34 | 320.76 | 38.84 |
| **Sepsis** | German (DE), Central | 15 | 244.27 | 26.00 | 4.87 | 2.00 | 251.13 | 32.87 |
| **Controls** | German (DE), North | 93 | 230.04 | 26.12 | 4.73 | 2.34 | 237.12 | 33.19 |
| **ESP** | USA, European ancestry (EA) | appr. 4,200 | 251.06 | 26.90 | 5.12 | 2.15 | 258.33 | 34.17 |
| **ExAC** | Non-Finnish Europeans (NFE)  incl.US,GB,DE,SE | appr. 30,000 | 237.55 | 24.47 | 5.27 | 2.71 | 245.52 | 32.44 |

1000G: 1000 Genomes Project; ExAC: Exome Aggregation Consortium; ESP: NHLBI Exome Sequencing Project; US: United States of America, GB: Great Britain, DE: Germany, SE: Sweden.

**Table S6**

Results of Sanger sequencing validating randomly selected SNVs.

(see SupplementaryTables.xls)

**Table S7**

Rare deleterious SNVs in sepsis patients, affecting genes of 6 pathways and chr15q26 identified by LOOCV experiments;

3fm: 3fold damaging missense, st-g=stop-gained, sp-a: splice-acceptor, sp-d=splice donor; GalphaQ: REACTOME G ALPHA Q SIGNALLING EVENTS DetStim: DETECTION OF STIMULUS CDC42: PID CDC42 PATHWAY TOLL=BIOCARTA TOLL PATHWAY HER2: BIOCARTA HER2 PATHWAY: REACTOME FAcCoA=FATTY ACYL COA BIOSYNTHESIS.

| **Group** | **source pathway** | **gene** | **sample** | **pos_hg19** | **ExAC NFE (MAF)** | **ESP EA (MAF)** | **rs ID** | **ref** | **alt** | **type** | aa change |
| --- | --- | --- | --- | --- | --- | --- | --- | --- | --- | --- | --- |
| GR-A | GalphaQ | ADRA1D | GRA 18 | 20_4202574 | 0.040% |  | rs55926349 | G | A | 3fm | ARG/TRP |
|  |  | AGT | GRA 09 | 1_230846446 | 0.316% | 0.267% | rs61731497 | A | G | 3fm | CYS/ARG |
|  |  | DGKD | GRA 10 | 2_234377185 | 0.054% | 0.035% | rs139085833 | C | T | 3fm | ARG/TRP |
|  |  | DGKH | GRA 06 | 13_42733400 |  |  | . | A | T | sp-a |  |
|  |  | DGKH | GRA 06 | 13_42733401 |  |  | . | G | T | sp-a |  |
|  |  | EDNRB / EDNRB-AS1 | GRA 27 | 13_78472364 |  |  | . | G | A | 3fm | ARG/CYS |
|  |  | F2 | GRA 19 | 11_46741296 |  |  | . | C | T | 3fm | ARG/TRP |
|  |  | F2R | GRA 10 | 5_76029285 | 0.033% | 0.023% | rs2227799 | C | A | 3fm | SER/TYR |
|  |  | GNA14 | GRA 21 | 9_80262613 | 0.435% | 0.268% | rs138686336 | G | A | 3fm | ARG/CYS |
|  |  | GNA14 | GRA 32 | 9_80262613 | 0.435% | 0.268% | rs138686336 | G | A | 3fm | ARG/CYS |
|  |  | GPR132 | GRA 14 | 14_105517528 | 0.018% | 0.023% | rs200738332 | G | A | 3fm | ARG/CYS |
|  |  | GPRC6A | GRA 10 | 6_117127906 |  |  | rs200347739 | C | A | 3fm | GLY/VAL |
|  |  | KISS1R | GRA 27 | 19_917630 |  |  | . | G | A | st-g | TRP/stop |
|  |  | NMS | GRA 22 | 2_101093730 | 0.000% | 0.012% | rs368460081 | G | A | sp-d |  |
|  |  | NTSR2 | GRA 32 | 2_11810166 | 0.000% |  | rs186016078 | C | T | st-g | TRP/stop |
|  |  | P2RY2 | GRA 24 | 11_72945588 | 0.000% |  | . | C | G | 3fm | SER/ARG |
|  |  | PRKCQ | GRA 06 | 10_6472852 |  |  | . | G | A | 3fm | ARG/CYS |
|  |  | PROKR2 | GRA 30 | 20_5283033 |  |  | . | G | A | 3fm | ARG/CYS |
|  |  | TACR3 | GRA 24 | 4_104640490 | 0.000% |  | rs201886341 | G | A | 3fm | ARG/CYS |
|  |  | TRPC7 | GRA 31 | 5_135692762 | 0.002% |  | . | C | G | 3fm | ARG/PRO |
|  |  | P2RY11/PPAN-P2RY11 | GRA 18 | 19_10224443 | 0.027% | 0.012% | rs200236974 | C | T | 3fm | ARG/CYS |
|  | DetStim | ABCA4 | GRA 03 | 1_94512566 | 0.002% | 0.012% | rs61749446 | G | A | 3fm | ARG/TRP |
|  |  | GRM6 | GRA 29 | 5_178413523 | 0.253% | 0.210% | rs62638210 | G | A | 3fm | ARG/CYS |
|  |  | NLRC4 | GRA 08 | 2_32476005 | 0.028% | 0.070% | rs199475953 | G | A | st-g | ARG/stop |
|  |  | NLRC4 | GRA 14 | 2_32476376 |  |  | . | C | T | st-g | TRP/stop |
|  |  | NOD1 | GRA 28 | 7_30492344 |  |  | . | A | C | 3fm | PHE/CYS |
|  |  | RP1 | GRA 05 | 8_55533893 | 0.003% |  | . | C | T | 3fm | ARG/CYS |
|  |  | TIMELESS | GRA 01 | 12_56814414 | 0.001% |  | . | T | A | 3fm | GLU/VAL |
|  |  | UNC119 | GRA 11 | 17_26874326 | 0.003% | 0.012% | rs143353275 | C | T | 3fm | GLY/ARG |
|  | CDC42 | BCAR1 | GRA 07 | 16_75263822 | 0.002% |  | . | C | G | 3fm | GLY/ARG |
|  |  | LIMK1 | GRA 12 | 7_73521431 | 0.002% |  | . | C | T | 3fm | ARG/CYS |
|  |  | MTOR | GRA 15 | 1_11272478 | 0.036% | 0.023% | rs151082401 | T | C | 3fm | TYR/CYS |
|  |  | TIAM1 | GRA 21 | 21_32492772 | 0.004% | 0.012% | rs372955793 | C | T | 3fm | GLY/ARG |
|  |  | TNK2 | GRA 02 | 3_195595559 | 0.151% | 0.192% | rs143787673 | C | A | 3fm | SER/ILE |
|  | TOLL | TLR10 | GRA 18 | 4_38774898 | 0.212% | 0.116% | rs145139818 | G | A | st-g | ARG/stop |
|  |  | TLR10 | GRA 16 | 4_38774898 | 0.212% | 0.116% | rs145139818 | G | A | st-g | ARG/stop |
|  |  | TOLLIP | GRA 17 | 11_1298412 | 0.037% | 0.012% | rs144425237 | G | A | 3fm | ARG/CYS |
|  | TOLL+CDC42 | MAP3K1 | GRA 06 | 5_56160642 |  |  | . | C | T | 3fm | ARG/CYS |
|  | HER2 | ERBB3 | GRA 23 | 12_56495039 |  |  | . | C | G | st-g | TYR/stop |
|  |  | IL6R | GRA 05 | 1_154401858 | 0.017% | 0.023% | rs145766309 | C | T | 3fm | SER/PHE |
|  | FAcCoA | ACLY | GRA 08 | 17_40039467 | 0.236% | 0.360% | rs79062040 | A | C | 3fm | TYR/ASP |
|  |  | ACLY | GRA 13 | 17_40039467 | 0.236% | 0.360% | rs79062040 | A | C | 3fm | TYR/ASP |
|  |  | ACLY | GRA 19 | 17_40039467 | 0.236% | 0.360% | rs79062040 | A | C | 3fm | TYR/ASP |
|  |  | ACLY | GRA 26 | 17_40039467 | 0.236% | 0.360% | rs79062040 | A | C | 3fm | TYR/ASP |
|  |  | ELOVL5 | GRA 04 | 6_53160466 |  |  | . | T | C | 3fm | TYR/CYS |
|  | chr15q26 | AP3S2/C15orf38-AP3S2 | GRA 01 | 15_90431823 |  |  | . | T | C | 3fm | TYR/CYS |
|  |  | KIF7 | GRA 27 | 15_90173634 | 0.073% | 0.035% | rs147191956 | G | A | 3fm | ARG/TRP |
|  |  | LRRC28 | GRA 21 | 15_99892588 | 0.003% |  | . | C | T | st-g | ARG/stop |
|  |  | LYSMD4 | GRA 12 | 15_100271946 | 0.050% | 0.047% | rs201005514 | G | C | 3fm | SER/ARG,LEU/VAL |
|  |  | LYSMD4 | GRA 20 | 15_100271946 | 0.050% | 0.047% | rs201005514 | G | C | 3fm | SER/ARG,LEU/VAL |
|  |  | MESP1 | GRA 25 | 15_90294153 | 0.006% |  | . | C | A | st-g | GLU/stop |
| GR-B | GalphaQ | EDNRB/EDNRB-AS1 | GRB 15 | 13_78472408 |  | 0.012% | rs200548885 | G | A | 3fm | SER/LEU |
